# Interpretation and use of a decision support tool for multiple treatment options: a combined randomised controlled trial and survey of medical students

**DOI:** 10.1136/bmjebm-2023-112370

**Published:** 2023-10-13

**Authors:** Birk Stokke Hunskaar, Per Olav Løvsletten, Ashley Muller, Per Olav Vandvik

**Affiliations:** 1 Institute of Health and Society, University of Oslo Faculty of Medicine, Oslo, Norway; 2 Department of Medicine, Lovisenberg Diakonale Hospital, Oslo, Norway; 3 Norwegian Centre for Addiction Research, University of Oslo Faculty of Medicine, Oslo, Norway; 4 Sørlandet sykehus HF Kristiansand, Kristiansand, Norway

**Keywords:** Evidence-Based Practice, Systematic Reviews as Topic, Clinical Decision-Making

## Abstract

**Objectives:**

To investigate medical students’ ability to interpret evidence, as well as their self-assessed understandability, perceived usefulness and preferences for design alternatives in an interactive decision support tool, displaying GRADE evidence summaries for multiple treatment options (Making Alternative Treatment CHoices Intuitive and Trustworthy, MATCH-IT).

**Design:**

A combined randomised controlled trial and survey. Participants were presented with a clinical scenario and randomised to one of two versions of the MATCH-IT tool (A/B), instructed to explore the evidence and decide on a recommendation. Participants answered a questionnaire assessing interpretation, treatment recommendation self-assessed understandability and perceived usefulness before exposure to the other MATCH-IT version and asked questions on design preferences.

**Setting:**

Online lecture in an evidence-based medicine (EBM) introductory course.

**Participants:**

149 third-year medical students. 52% (n=77) had 6 months of clinical training and 48% (n=72) had preclinical training only.

**Interventions:**

The MATCH-IT tool version A uses colour coding to categorise interventions by magnitude and direction of effects and displays all outcomes in a table on entry. Version B has no colour coding, and the user must decide which outcomes to display in the table.

**Main outcome measures:**

Interpretation of evidence, treatment recommendation, perceived usefulness and understandability, preference for format and design alternatives.

**Results:**

82.5% (n=123) of medical students correctly answered ≥4 out of 5 multiple choice questions assessing interpretation of data. 75.8% (n=114) of students made a treatment recommendation in accordance with an expert panel for the same clinical scenario. 87.2% (n=130) found the tool understandable while 91.9% perceived the tool as useful in addressing the clinical scenario.

**Conclusion:**

Medical students with no prior training in EBM can interpret and use the MATCH-IT tool. Certain design alternatives were preferred but had no bearing on interpretation of evidence or understandability of the tool.

**WHAT IS ALREADY KNOWN ON THIS TOPIC**

- Clinicians need point-of-care decision support tools to understand and balance benefits and harms, including multiple treatment options based on complex evidence from network meta-analysis.
- Practice of evidence-based medicine (EBM) has shifted from critical appraisal of the literature towards efficient use of EBM resources and tools.
- Little is known about how healthcare professionals and trainees can understand and employ such tools.

**WHAT THIS STUDY ADDS**

- Medical students can likely understand interactive GRADE evidence summaries for multiple treatment options, without prior training.
- Medical students prefer categorisation of results through colour coding when interacting with such complex evidence.

**HOW MIGHT THIS STUDY AFFECT RESEARCH, PRACTICE, OR POLICY**

- We hypothesise that also residents and attending doctors can understand and use interactive GRADE evidence summaries (interactive summary of findings tables) for multiple treatment options.
- Employment of an EBM-based decision support tool appears promising as a way of introducing students to EBM.

## Introduction

In what has been labelled a medical misinformation mess, healthcare professionals frequently struggle with finding, interpreting and applying the best current evidence for patient care.^1^ Adding to this struggle is the emergence of comparative effectiveness research with network meta-analysis (NMA) allowing comparisons of multiple treatment options using both direct and indirect evidence.^2^ In acknowledgement of the fact that most clinicians will never have the in-depth skills in critical appraisal required (or the time to apply them) to guide their clinical practice,^3^ there has been a shift towards training clinicians in how to find and appropriately use evidence-based medicine (EBM) tools and resources, decision support tools among them.^4^ Consequently, a key solution to assist healthcare professionals in coping with the misinformation mess and providing EBM-guided care to their patients is to provide available trustworthy decision support tools to doctors and patients alike.^1^

Trustworthy clinical practice guidelines constitute the preferred tools for decision making for medical professionals. According to standards for trustworthy clinical practice guidelines, such tools need to describe both benefits and harms of all treatment options with an associated certainty, based on evidence summaries from systematic reviews.^5^ Structured evidence summaries providing such information now frequently appear in guidelines and systematic reviews, using Grading of Recommendations, Assessment, Development, and Evaluation (GRADE) methods.^6^ These are also available in interactive formats (GRADE interactive Summary of Findings, iSoF).^7^ As future doctors and practitioners of EBM, medical students ought to be taught EBM in line with recent developments and practice. Despite the conceptual shift in the practice of EBM and the advent of NMA, decision support tools for multiple treatment options remain a rare breed, and we are not aware of any studies examining the use of tools fulfilling these standards on medical students; neither in an educational context nor if they are able to at all understand and use such tools.

Acknowledging these shortcomings, decision support tools displaying interactive GRADE evidence summaries for multiple treatment options are being developed.^8^ One such tool is the Making Alternative Treatment CHoices Intuitive and Trustworthy (MATCH-IT) tool.^9^ Whereas MATCH-IT tools have been published within the context of trustworthy guidelines^10^ there is a need to further test, evaluate and improve how this tool works for different target groups. For this purpose, we are currently doing both qualitative user testing research as well as surveys and trials directed at a variety of target groups. In this study, we tested an early prototype of the MATCH-IT tool in an educational context on medical students without prior training in EBM and little or no clinical experience. We asked: (1) To what extent do medical students correctly interpret GRADE evidence summaries as presented in MATCH-IT, and what treatment would they recommend in the clinical scenario? (2) To what extent is MATCH-IT perceived as useful and understandable in addressing the clinical scenario, in an educational context? (3) Is there a preference for one version of MATCH-IT over the other?

## Methods and materials

### Overview

**Figure 1:**
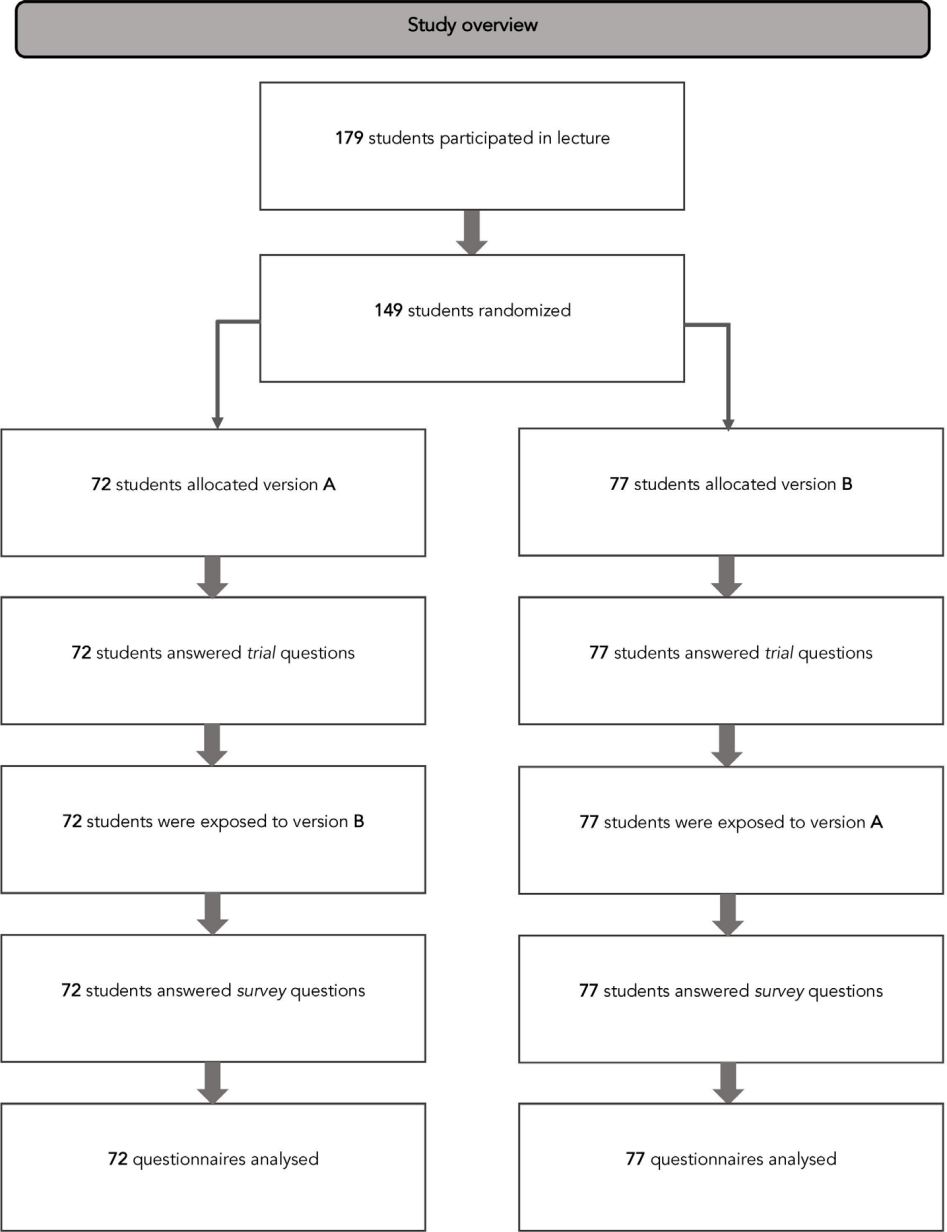
Flow chart of study.

The study was performed as a joint lecture and study conducted by a medical student in a research curriculum (BSH) and his supervisor (POV) over Zoom due to COVID-19 restrictions. We applied a combined RCT and crossover survey design. In the lecture, the students were presented with a clinical scenario and questions regarding best current diabetes drugs management (box 1). The students were then randomised to interact with one of two versions of the MATCH-IT tool and answered multiple choice questions in an online questionnaire. The questions assessed their interpretation of the evidence, their treatment recommendation for the clinical case, and whether the tool was perceived as useable and understandable by the students. After exposure to the other version of the tool, students answered questions about their individual preferences for specific design alternatives. Figure 1 shows an illustration of the study.

Box 1 **Description of clinical scenario**

Setting: general practice

Objective: To consider appropriate medical management for a 64-year-old man with type II diabetes and established cardiovascular and renal disease, currently only treated with metformin for glucose management.

Your patient has recently stumbled across information that SGLT2-inhibitors might be a medication to add for a patient like him with his cardiovascular risk profile. Now he wants to discuss this with you. In preparation for the clinical encounter, you came across a new guideline in *BMJ* on the use of GLP-1 receptor analogues and SGLT2-inhibitors in type II diabetes. The guideline highlights a link to a decision support tool, the Making Alternative Treatment CHoices Intuitive and Trustworthy tool, that allows you to explore the evidence.

Use the tool to familiarise with the evidence and to consider if you would recommend adding one of the drugs to your patient. If so, which one?

### Population and setting

The study was conducted in 4 January 2021, at the beginning of a 1-week introductory course in EBM for third-year medical students from two classes in University of Oslo. One class had 6 months of clinical training, the other had preclinical training only. Due to COVID-19 restrictions students were not physically present and attended via Zoom.

As part of first year preclinical training, all students had completed and passed the exam on the introductory course in biostatistics. As such, the students had previously been introduced to key concepts such as absolute and relative risk and CIs. The students had not yet been introduced to more EBM focused concepts such as PICO questions, certainty of the evidence or GRADE as part of their medical studies.

In a short introduction of the study, we gave the students a brief recap on diabetes management principles in general. We informed about the aim of the study and gathered informed consent. Finally, we introduced the clinical scenario before they were asked to open the online questionnaire (box 1 presentation can be viewed as online supplemental file 1).

10.1136/bmjebm-2023-112370.supp1 Supplementary data

### Randomisation and questionnaire

After students had entered the online questionnaire and registered to which class they attended, they were automatically randomised by the online questionnaire service to interact with either version A or B of the MATCH-IT tool. The questionnaire service^11^ randomly allocates respondents using Java^12^ without investigators involvement in either sequence generation, allocation or implementation. As such, investigators and students were blinded as to which version they were allocated.

They were given a maximum of 8 min to interact with MATCH-IT (a reminder was given after 5 min). In this time, they were asked to answer five multiple-choice questions about the evidence, provide a treatment recommendation and rate the perceived usefulness and their self-assessed understandability of the tool (see section on the Outcome measures for further discussion, full questionnaire can be viewed as online supplemental file 2.

10.1136/bmjebm-2023-112370.supp2 Supplementary data

The students were then given access to the other version of the MATCH-IT tool and asked to interact with it for maximum 5 min before rating their overall preference for either MATCH-IT tool version A or B, in addition to their preference for two key design features.

The initial questionnaire was created by BSH and then iteratively revised and reviewed by authors (BSH, POL and POV) and the wider research team (see the Acknowledgments section) until a consensus was made on a final version.

### Presentation formats

The MATCH-IT tool is an iSoF table for multiple treatment options that presents GRADE evidence summaries from NMAs. It allows interactive display of multiple outcomes across all interventions, for which the users can shuffle the order and change number of comparisons. For every comparison, absolute effects for dichotomous outcomes are presented as risk differences (number of cases per 1000 patients treated for a certain period) and GRADE certainty ratings are displayed. The tool also includes a practical issues module, and it is possible to display a pictogram of pairwise comparisons in a pop-up window. For these features, the students were neither prompted to use them nor had to use them to answer the questionnaire correctly.

**Figure 2:**
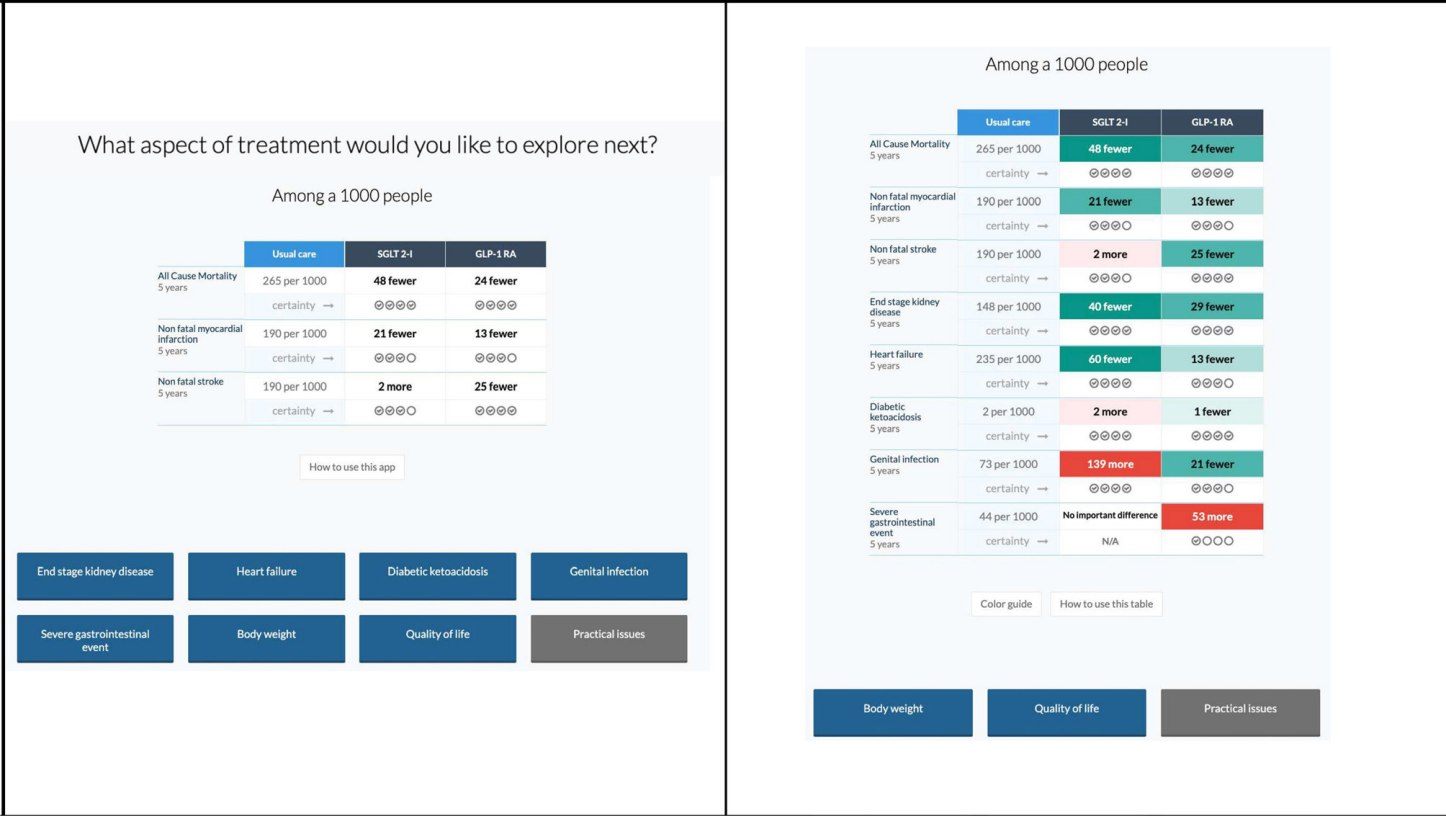
Left: Version A with colour coding and full table open on entry. Right: Version B with no colour coding, and outcomes had to be clicked to be shown in the table.

Screenshots of the two versions of the MATCH-IT tool (A and B) tested in this study are displayed in figure 2, and can be accessed and interacted with through these links: MATCH-IT A and MATCH-IT B. The versions differed in two design features. MATCH-IT A uses (a) colour coding to categorise interventions by magnitude and direction of effects and (b) displays the results for all outcomes up front in an open table. MATCH-IT B uses (a) no colour coding or other ways to visually categorise interventions and (b) displays a closed table up front which allows students to choose which and how many outcomes to display.

### Outcome measures

Interpretation of evidence was measured using five multiple choice questions. Each question had 4–6 response categories with 1–2 correct answers. We also measured the proportion of students that recommended treatment in congruence with the trustworthy guideline; the panel issued a strong recommendation for adding SLGT-2 inhibitors to standard therapy for patients in this risk group.^13^ The students were at no point informed of this recommendation.

We measured perceived usefulness and self-assessed understandability of the tool using 7-point Likert scale ranging from ‘strongly disagrees to ‘strongly agree’ with a neutral option in the middle, corresponding to ratings 1–7.

Overall preference for MATCH-IT tool A or B and preference for design alternatives (ie, preference for colour coding vs no colour coding; and preference for displaying an open full table vs a closed table on entry) was measured using a 7-point Likert scale ranging from ‘strong preference for A’ to ‘strong preference for B’. Ratings 1–3 were in favour of version B, rating 4 was indifference between the two versions and 5–7 were in favour of version A. See online supplemental file 2 for the full questionnaire with questions and response alternatives.

### Statistical analysis

We planned to report aggregated results for the whole sample in addition to comparison of the randomised groups. For data assessing interpretation of evidence, we created a composite variable of responses to multiple choice questions. Here, we pragmatically defined four out of five correct answers to constitute correct overall interpretation. For the ordinal variables (perceived usefulness and self-assessed understandability, preferences), we visually inspected the distributions. These distributions were all either U-shaped or clustered strongly towards one side. For the perceived usefulness and self-assessed understandability variables, we, therefore, dichotomised to useful/not useful and understandable/not understandable. For the treatment recommendation variable and preference variables, we wanted to capture the proportion of students who provided no treatment recommendation or who had no preference. We, therefore, divided these variables into three categories.

To compare the two randomised groups, we used the Mann-Whitney U non-parametric test for outcomes with ordinal variables that had three or more categories. We used χ^2^ tests for dichotomised outcomes. We used STATA for all statistical analysis.

We did not perform a formal power calculation to detect a statistically significantly difference a priori. We knew that approximately 200 students would attend the lecture and hypothesised that at least 140 students would participate in the study.

## Results

### Participants

Of 230 students in the two classes, 78% (n=179) attended the lecture, of whom 84% (n=149) participated in the study, with no dro-pouts. The 17% (n=30) who did not participate did not access the link once it was shared.

Of the 149 participating students, 48% (n=72) students were randomised to version A and 52% (n=77) students to version B. Fifty-two per cent (n=77) students had undergone 6 months of clinical training while 48% (n=72) had preclinical training only.

### Understanding and correct interpretation

**Figure 3:**
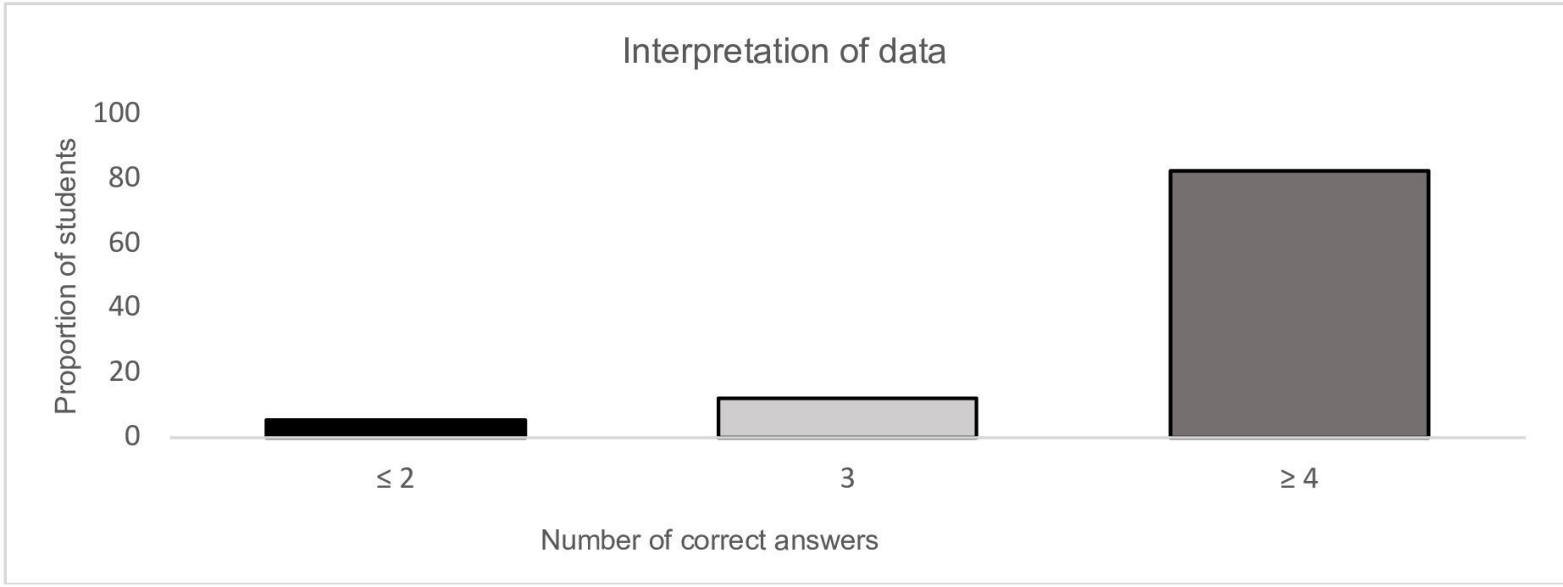
Bar chart of distribution of correct answers.

Figure 3 shows the distribution of correct answers to the five questions, with 123 students (82.6%) having ≥4 of 5 correct answers. The number of correct answers did not differ according to whether the students were first randomised to version A or B (p=0.42), see table 1 for all data from trial portion of study.

**Table 1:** Data from trial portion of study

Trial data				
Outcome	Entire sample N (%)	Version A N (%)	Version B N (%)	Test statistic
Understanding and correct interpretation				Z=−1.351, p=0.4178
5/5 correct answers	74 (49.7)	35 (45.5)	39 (54.2)	
4/5 correct answers	49 (32.7)	25 (32.3)	24 (33.3)	
3/5 correct answers	18 (12.1)	12 (15.5)	6 (8.3)	
2/5 correct answers	5 (3.4)	4 (5.2)	1 (1.39)	
1/5 correct answers	2 (1.3)	1 (1.3)	1 (1.39)	
0/5 correct answers	1 (0.7)	0	1 (1.39)	
Perceived usefulness of MATCH-IT				χ2 (1, 149)=1.7588, p=0.185
Useful or very useful	137 (91.9)	73 (94.8)	64 (88.9)	
Not useful	12 (8)	4 (5.2)	8 (11.1)	
Perceived understandability of MATCH-IT				χ2 (1, 149)=0.1620, p=0.687
Understandable or very understandable	130 (87.2)	68 (88.3)	62 (86.1)	
Not understandable	19 (12.8)	9 (11.7)	10 (13.9)	
Treatment recommendation using MATCH-IT				Z=0.197, p=0.844
SGLT2-inhibitors	114 (75.8)	58 (75.3)	56 (77.7)	
Different treatment recommendation	19 (12.8)	12 (15.6)	7 (9.7)	
No treatment recommendation	16 (10.7)	7 (9)	9 (12.5)	

MATCH-IT, Making Alternative Treatment CHoices Intuitive and Trustworthy.

### Correct treatment recommendation after using MATCH-IT

The proportion of students recommended treating with SLGT-2 inhibitors in the clinical case was 75.8% (n=113), 12.8% (n=19) chose a treatment recommendation other than SGLT2, whereas 10.7% (n=16) chose not to provide a recommendation. There was no statistically significant difference between the groups’ recommendations (p=0.844).

### Perceived usefulness and self-assessed understandability of MATCH-IT

The proportion of students that perceived the tool useful or very useful in solving the clinical case was 91.9% (n=137). The proportion of students finding the tool ‘useful or very useful’ compared with ‘not useful’ did not differ according to the version the students were first randomised to (p=0.185).

A similarly high proportion, 87.4% (n=130) found the tool understandable or very understandable. Reporting understandability did not differ according to randomisation to version A or version B (p=0.687).

### Preferences for MATCH-IT version and specific design features

**Figure 4:**
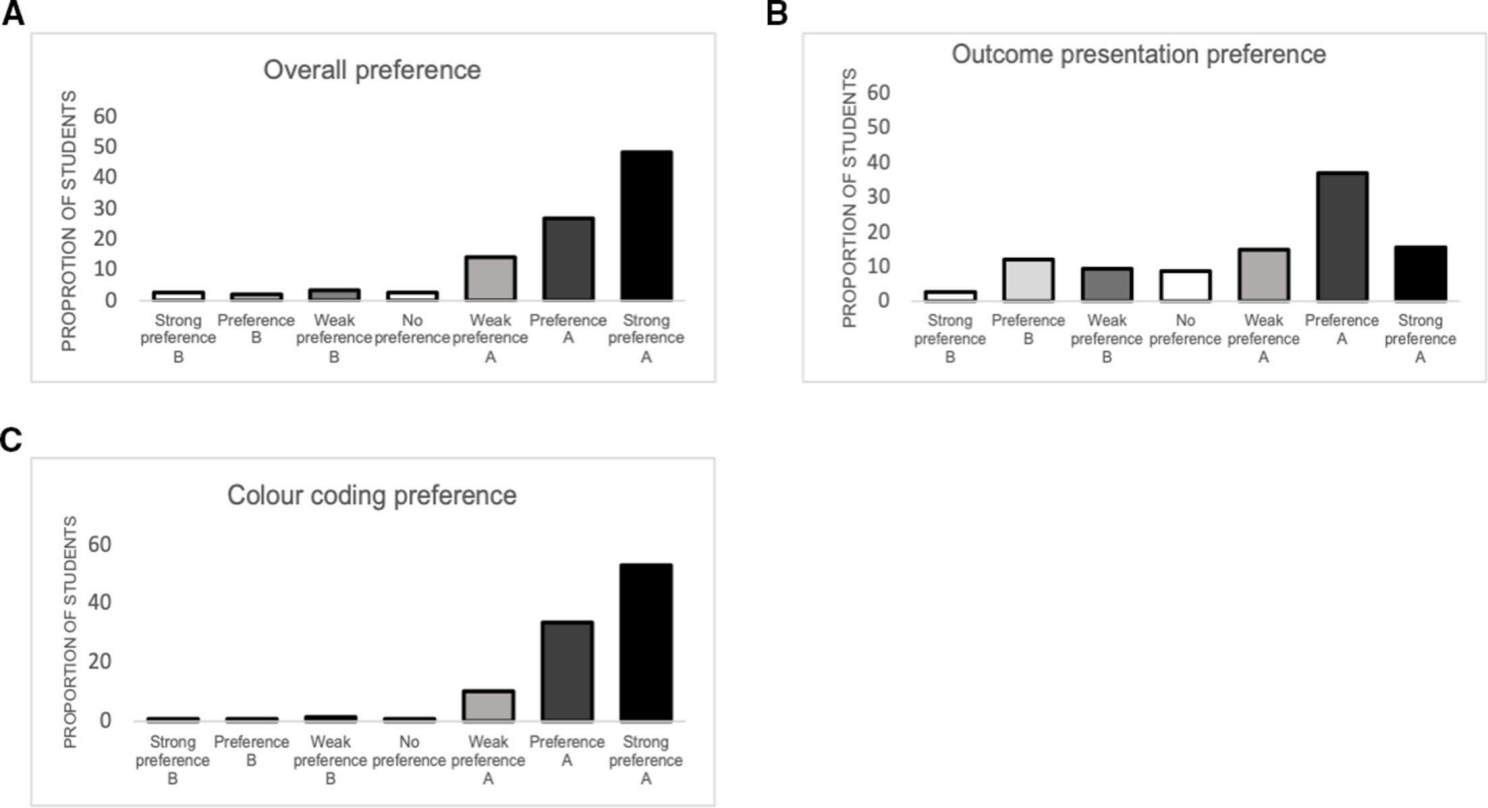
Overall preference for format (A), preference for presentation of outcomes (B) and preference for colour coding (C)*.*

After exposure to both formats of the MATCH-IT tool, 86.3% (n=133) of students reported a preference for version A. Concerning initial display of outcomes, 67.1% (n=100) of students preferred having all outcomes displayed on entry (version A) while 32.9% (n=49) students either preferred having to select outcomes (version B) or showed no preference at all. Concerning colour coding to reflect direction and magnitude of effects, 96.6% (n=144) of students reported a clear preference for colour coding (version A), with only 3.4% (n=3) of students reporting preference for the table without colour coding. Across all preference outcomes, there was no significant difference in preference whether you were randomised to version A or B first. Data on preferences is displayed in figure 4. All data from survey portion can be viewed in table 2.

**Table 2:** Survey data

Outcome	Entire sample N (%)	Version B N (%)	Version A N (%)	Test statistic
Version preference				Z=1.765, p=0.0775
Version B	12 (8)	3 (3.9)	9 (12.5)	
Version A	133 (86.26)	72 (93.5)	61 (84.7)	
No preference	4 (2.7)	2 (2.6)	2 (2.7)	
Outcome preference				Z=−0.627, p=0.5253
** **Select the outcomes (version B)	36 (24.2)	18 (23.4)	18 (25)	
** **All outcomes displayed (version A)	100 (67.1)	49 (63.6)	51 (70.8)	
** **None	13 (8.7)	10 (13)	3 (4.2)	
Colour coding preference				Z=0.518, p=0.9363
No colour coding (version B)	4 (2.7)	2 (2.6)	2 (2.7)	
Colour coding (version A)	144 (96.6)	75 (97.4)	69 (95.8)	
None	1 (0.7)	0 (0)	1 (1.4)	

## Discussion

### Main findings

Our study showed that most medical students can understand and use the MATCH-IT tool—an iSOF for multiple treatment options based on evidence from NMA. The use of colour coding to communicate direction and magnitude of effect was clearly preferred by the students. In terms of clinical decision-making, a large majority of students was able to reach the same treatment decision for the specified population as an international guideline panel comprised of experts.^13^ Colour coding was likely the primary driver of preference towards version A of the MATCH-IT tool, although students also preferred having all outcomes shown on entry of the tool as opposed to choosing on their own which outcomes to view. There was no statistically significant difference between the groups for any outcome. Thus, the design features had no bearing on whether the students correctly interpreted the evidence or regarded the tool as useful or understandable.

### This study and current research

The present study is part of the MATCH-IT project^9^—a combined research and software development project employing user testing and software development iteratively develop a presentation format for NMA data. Due to the combined nature of the project, the rationale for conducting the study was manyfold. In addition to testing whether such a tool was at all understandable and useable by healthcare trainees, the study presented an opportunity for the MATCH-IT team to pilot quantitative research methods to be used in further evaluations, where we aim to test the tool against other presentation formats for NMA data.^14–16^ At the time of conducting the study, the MATCH-IT team had yet to decide on which of the design features highlighted in the study to use in further development. Thus, the present results were—together with results from qualitative user testing studies—used to make design decision.

Although our study was done on medical students, we hypothesise that healthcare professionals with clinical experience, such as medical doctors, will find the tool equally or even more easy to use and understand in clinical decision-making. However, as medical students are relatively untrained and unfamiliar in clinical decision-making processes, they may have different needs as users and may be unable to identify key issues and points of confusion that may bother other audiences such as practicing clinicians. Consequently, further research is needed to corroborate the generalisability of our study.

While a wide range of presentation formats for NMA have been developed over the last decade,^17^ as well as some recent emerging decision-support tools beyond MATCH-IT,^14–16^ we have not identified any studies examining the usability or understandability of these presentation formats by healthcare trainees or professionals. One recent study^18^ applied structured qualitative research methods iteratively to guide the development of a static tabular GRADE evidence summary presentation format. Like version A of the tool—which was preferred by the students—the creators used colour coding to signal direction and magnitude of effect, strengthening the hypothesis that colour coding is preferable when presenting results from NMAs.

Our study tested a decision support tool on medical students in an educational setting. As mentioned in the introduction, EBM practice and education is undergoing a conceptual shift towards intelligent use of EBM resources and tools, in acknowledgement of the practical problems associated with critical appraisal of literature at the point of care.^4^ Current research in EBM education centres largely around the comparative effectiveness of alternative teaching strategies,^19 20^ and we have identified no studies describing or examining the use of EBM resources and tools in an educational context. Thus, it does not seem like current EBM education research reflects this shift. Although the students were able to successfully use and understand the tool, measuring postintervention effects such as skills, knowledge, attitudes and behaviours—as is the norm in EBM education research^19 21 22^—was outside the scope of our study.

### Strengths and limitations

The present study included 149 participants and applied methodological measures to limit risk of bias (randomisation to intervention arm, blinding of participants). A major limitation of our study is the lack of a third arm, exposing the medical students either to ‘no intervention’ or to a conceptually different visualisation tool for NMA results.^14–16^ Even though the versions of MATCH-IT employed in the study differed in certain design elements, they are conceptually and structurally similar; they are tabular formats, iSoFs and employ many of the same design features. At the time of conducting our study, many now relevant—and possibly comparable—presentation formats such as the Vitruvian plot^15^ and the Kilim plot^14^ had yet to be published, while time constraints kept us from being able to create an existing presentation format—such as the rank-heat plot^16^ presenting the same data used in the study. As mentioned, we aim to evaluate the tool against other presentation formats at a later stage.

Another limitation to the generalisability of our study is that we have only examined the research questions using a single clinical question with a corresponding decision support tool—MATCH-IT for type II diabetes drugs. This decision support tool, published as part of a clinical practice guideline,^13^ includes 3 interventions and 10 outcomes. Thus, the generalisability of the results with regards to more complex bodies of evidence with more interventions and outcomes may be limited. Our results would have been strengthened by a more comprehensive user testing, reflecting a wider variability of clinical questions and more complex evidence. Such user testing has been performed in the qualitative part of the MATCH-IT project, where we have tested datasets across several different topics containing up to 27 interventions and 9 outcomes.^23^ The results from these studies generally support our findings and will be published over the coming year. One may also question the use of self-assessment to assess the true usefulness and understandability of the tool, although this is common in educational research.^20^ We tried to corroborate this by objectively assessing interpretation of the presented evidence as well, rather than using self-assessed variables only.

A discussion of the validity of the questionnaire and questionnaire items is warranted. Using Nemoto and Beglar^24^ as a guide, the present questionnaire can be examined for both strengths and weaknesses. In favour of the validity of the questionnaire, it was created through several revisions, being reviewed by experienced researchers and teachers in the field of EBM before arriving at a final version. Further, the questionnaire items are unambiguous and written in straightforward, familiar language and assess only one concept/idea per item. The present study uses 7-point Likert scales with a neutral option, as opposed to the recommended 6-point scale without a neutral option. For scales assessing preference, we wanted to include a neutral option as it could be reasonably expected that respondents might not prefer either version. Adding to this, part of the rationale for excluding neutral options in Likert scales pertains to the problems it causes when analysed using formal measurement models such as Rasch models^25^; we did not use or plan to use a formal measurement model for analysis of the questionnaire data.

## Conclusion

We conclude that interactive GRADE evidence summaries for multiple comparisons—here represented by the MATCH-IT tool—can be understood and used by medical students. The application of colour coding is preferable, but not necessary, for medical students to understand such presentation formats. As such, we argue that creators of presentation formats for NMA should consider applying colour coding.

Although the students were able to understand and use the tool in this study, the current literature on the use of EBM tools and resources by healthcare trainees remains scarce both in terms of the feasibility of using these tools in an educational context and in terms of how such tools may influence postintervention EBM competencies: skills, knowledge, attitudes and behaviours. As EBM practice and education shifts towards intelligent use of resources and tools, we argue that further research in this field is needed.

## Data Availability

Data are available on reasonable request. All data relevant to the study are included in the article or uploaded as online supplemental information.
