# Supplementary material for: Interpretation and use of a decision support tool for multiple treatment options: a combined randomised controlled trial and survey of medical students
Source: BMJ Evid Based Med. 2023 Oct 13;29(1):29–36. doi: 10.1136/bmjebm-2023-112370 (PMC10850623; doi:10.1136/bmjebm-2023-112370)
Supplement: Supplementary data [file bmjebm-2023-112370supp001.pdf]

On well informed clinical decision making

The core of Evidence-Based Medicine and your future as a doctor

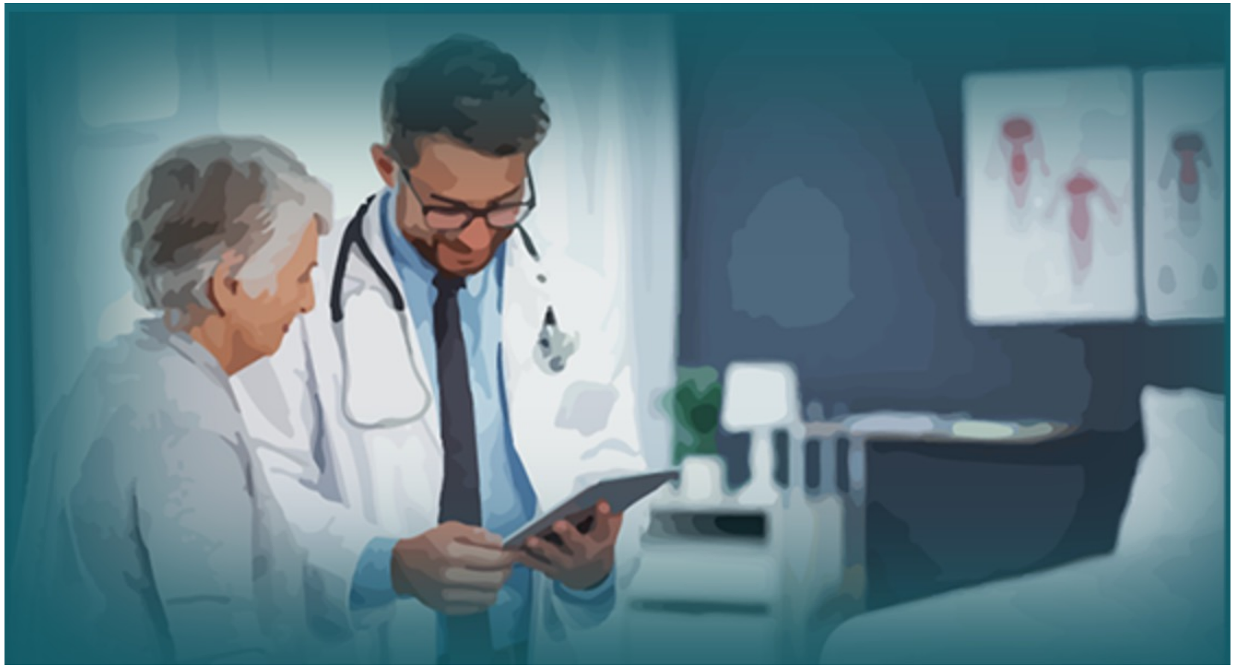

Senior physician Per Olav Vandvik LDS

Professor, Evidence Practice program, DMF-UiO

CEO, MAGIC Evidence Ecosystem Foundation

Type 2 Diabetes Mellitus

A pandemic with global and personal consequences

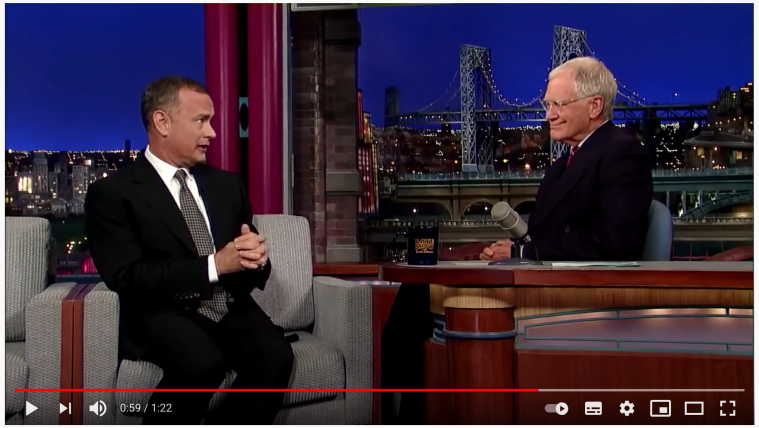

David Letterman Tom Hanks' Type 2 Diabetes

Treatment principles:

- **Life style interventions** (physical activity, dietary changes) are always tried, but seldom enough
- **Traditional medications** (such metformin, insulin) regulate the blood glucose, but have uncertain benefits besides this
- **New medications breaking through:** showing positive effects on cardiovascular disease and chronic kidney disease:
    - Sodium-glucose cotransporter-2 inhibitors (SGLT-I)
    - Glucagon-like peptide-1 receptor agonists (GLP1-RA)

- Which patients have the most benefits from these drugs, and how do we make decisions in clinical practice?

What effects do SGLT-2 and GLP-RA have for our patients?

SGLT2 inhibitors for primary and secondary prevention of cardiovascular and renal outcomes in type 2 diabetes: a systematic review and meta-analysis of cardiovascular outcome trials

Thomas A Zelniker, Stephen D Wiviott, Itamar Raz, Kyungah Im, Erica L Goodrich, Marc P Bonaca, Ofri Mosenzon, Eri T Kato, Avivit Cahn, Remo H M Furtado, Deepak L Bhatt, Lawrence A Leiter, Darren K McGuire, John P H Wilding, Marc S Sabatine

Summary
Background The magnitude of effect of sodium-glucose cotransporter-2 inhibitors (SGLT2i) on specific cardiovascular and renal outcomes and whether heterogeneity is based on key baseline characteristics remains undefined.

Lancet 2019; 393: 31–39
Published Online

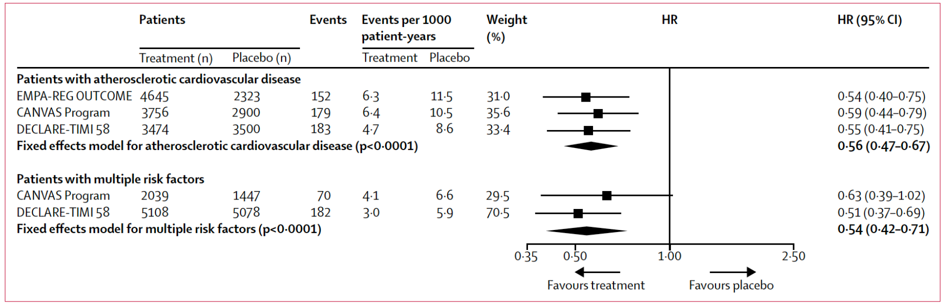

Figure 4: **Meta-analysis of SGLT2i trials on the composite of renal worsening, end-stage renal disease, or renal death stratified by the presence of established atherosclerotic cardiovascular disease**
Atherosclerotic cardiovascular disease: Q statistic=0·19, p=0·91, I^2=0%; multiple risk factors: Q statistic=0·52, p=0·47, I^2=0%. The p value for subgroup differences was 0·71. Tests for subgroup differences were based on F tests in a random effect meta-regression estimated using restricted maximum likelihood and Hartung Knapp adjustment. HR=hazard ratio. SGLT2i=sodium-glucose cotransporter-2 inhibitors.

- We need summarized research as a basis for well-informed clinical decisions.
- The picture shows relative effects on chronic kidney disease, but what about cardiovascular disease and adverse events?
- A new systematic review and NMA in BMJ 2021
  - 764 randomized studies with 420 000 patient.
  - Provides reliable data on benefits and harms with SGLT-2 and GLP1-RA.
- How do we present results in more understandable formats?
  - We need absolute effects for all relevant outcomes that matter for our patients
  - Learning goals in EBM are to find, appraise and apply such results in clincal practice

Why should you care about Evidence Based Medicine?

UiO : Universitetet i Oslo

MAGIC Evidence Ecosystem Foundation

MATCH-IT

Making **A**lternative **T**reatment **CH**oices **I**ntuitive and **T**rustworthy

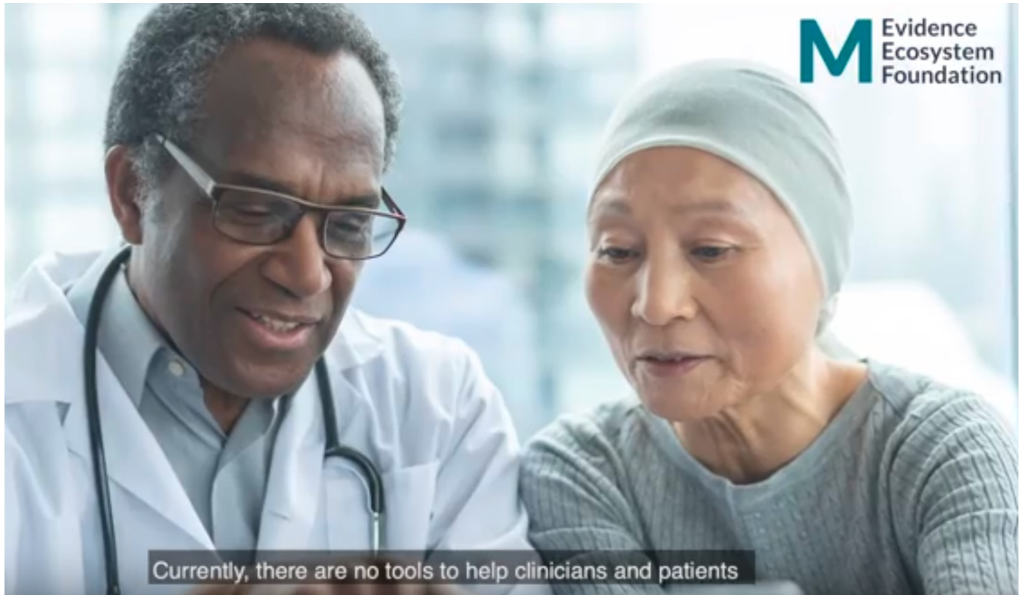

UiO : Universitetet i Oslo

MAGIC Evidence Ecosystem Foundation

What will happen now?

- We are developing a tool aiming to help professionals make well-informed clinical decisions
- This is a study that we want to publish.
- We do not gather any personal data. Participation is voluntary. You may refrain from participating if you wish
- Your participation will be highly appreciated.
- You will be introduced to a clinical scenario that we want you to address. To solve it you will use our decision support tool.

UiO : Universitetet i Oslo

MAGIC Evidence Ecosystem Foundation

Clinical scenario

- You are Per's, a 64 year old telephone salesman with diabetes type 2, GP. He has estabilshed cardiovascular disease and chronic kidney disease, but is in otherwise good general condition.
- He comes to you for a yearly checkup with regards to his diabetes.
- He has read about a new drug called SGLT2 inhibitors and he is wondering if he should be taking this drug in addition to metformin, which he is currently on.
- You know Per well, and knows that we wants to reduce the risk of further complications, such as death, myocardial infarction, heart failure, stroke and further kidney disease.
- You also know that there are two new drugs on the market: SGLT2-inhibitors and GLP1 analouges.
- With this information in mind, you wonder what is the best treatment for Per.

UiO : Universitetet i Oslo

MAGIC Evidence Ecosystem Foundation

MATCH-IT

- You check NEL (Norwegian Electronic Physicians handbook), but you find no recommendation pertaining to Per's situation. Using Google, you find a systematic review from the BMJ comparing these drugs with standard, traditional treatment (usual care). In the summary, you find a butten titled «decicion support». You press the button.

- Your task is to use the decision support tool to addres the clinical scenario: **What is the best treatment that you can provide for Per?**

UiO Universitetet i Oslo

MAGIC Evidence Ecosystem Foundation

MATCH-IT

- We are testing the **tool**, and not you!
- Still: please do your best when answering the questions.

Questionnaire

- You will access a questionnaire where there will be a link directing you to a tool. The tool will open inside your web browser.
- You will have 8 minutes in total to use the tool and answer 6 questions. A reminder will be given after 5 minutes, so that you don't forget to answer the questions.
- It is important to indentify the correct risk group for the patient.

UiO : Universitetet i Oslo

MAGIC Evidence Ecosystem Foundation

Choose baseline risk of diabetes

- Adults with no cardiovascular (CV) risk factors
- Adults with cardiovascular (CV) risk factors
- Adults with cardiovascular disease (CVD)
- Adults with chronic kidney disease (CKD)
- Adults with cardiovascular disease and chronic kidney disease (CVD+CKD)

UiO : Universitetet i Oslo

MAGIC Evidence Ecosystem Foundation

Summary of the case

- Per, 64 years old.
- Chronic cardiovascular disease
- Chronic kidney disease
- Wants to avoid further diabetes complications.
- You want to know if he should stay on usual care regime, or start with GLP1 analouges or SLGT2 inhibitors in addition.

- Your job is to find out what is the best treatment for Per.

UiO : Universitetet i Oslo

MAGIC Evidence Ecosystem Foundation

Plenary session

- Questions? Remarks?

UiO : Universitetet i Oslo

MAGIC Evidence Ecosystem Foundation
