# Supplementary material for: Interpretation and use of a decision support tool for multiple treatment options: a combined randomised controlled trial and survey of medical students
Source: BMJ Evid Based Med. 2023 Oct 13;29(1):29–36. doi: 10.1136/bmjebm-2023-112370 (PMC10850623; doi:10.1136/bmjebm-2023-112370)
Supplement: Supplementary data [file bmjebm-2023-112370supp002.pdf]

Appendix 2: Study questionnaire

Comments from the author:

- This questionnaire is a translation from the original Norwegian.
- Underscored answers signal which answers are correct for questions assessing interpretation.
-

Welcome to the study!

I attend class ____.

☐ Autumn 2018

☐ Spring 2019

---Randomization to different questionnaires for Version A and B was done at this point---

---

Hello!

You will now, using the MATCH-IT tool, decide on what is the best treatment for your patient, Per.

Please examine the effects of the treatments on the outcomes and answer some questions. For this task, you have 8 minutes allotted.

You do not have to use the full 8 minutes if you do not require or want to use them.

The study conductor will let you know when 5 minutes have passed. By this point, you should have started to answer the questions in the questionnaire.

Are you unsure of some of the details surrounding the case? A summary is shown in the Zoom-window.

Click the link below when you are ready to start.

MATCH-IT A: https://matchit.magicevidence.org/210104dist-diabetes-sof-open

MATCH-IT B: https://matchit.magicevidence.org/210104dist-diabetes-sof-closed

---

First impressions

Have you explored the evidence using the tool for 5 minutes or less? Great!

We want to gather your first impressions.

Consider the statements below and choose the option that most corresponds to your experience.

	Strongly disagree	Disagree	Somewhat disagree	Either or	Somewhat agree	Agree	Strongly agree
**It was easy to understand the information in the tool**	◯	◯	◯	◯	◯	◯	◯
**The tool was useful in addressing the clinical scenario**	◯	◯	◯	◯	◯	◯	◯

Questions about the treatments

We will now ask you some questions to assess your understanding of the information that was presented in the tool. It is possible to go back to the tool while answering the questions, but it is not required.

Which treatment reduces the risk of stroke the most?

- o GLP1-RA
- o SGLT2-I
- o Usual care
- o No difference
- o I don't know/remember/understand enough to answer the question

Which treatment reduces mortality the most?

- o GLP1-RA
- o SGLT2-I
- o Usual care
- o No difference
- o I don't know/remember/understand enough to answer the question

Do SGLT2 inhibitors (SLGT2-I) have any significant side effects?

- o Heart failure
- o Serious gastrointestinal adverse events
- o Genital infections
- o Weight change
- o I don't know/remember/understand enough to answer the question

Do GLP1 analogues (GLP1-RA) have any significant side effects?

- o Heart failure
- o Serious gastrointestinal adverse events
- o Genital infections
- o Weight change
- o I don't know/remember/understand enough to answer the question

Select the outcomes where there is little or negligible difference between the treatments.

- ☐ Body weight change
- ☐ Quality of life
- ☐ Chronic kidney disease
- ☐ Mortality
- ☐ Heart failure

Based on a consideration of all the outcomes, what is the best treatment for Per?

- o GLP1-RA
- o SGLT2-I
- o Usual care
- o No difference
- o I don't know/remember/understand enough to answer the question

*What was the reason you did not know, or were unable to answer the question? (*Shown only if previous answer was: "I don't know/remember/understand enough to answer the question")

Free text:

Use of the functions in the tool

Thank you for your participation so far! For the rest of the study, we have estimated 8 minutes. The study conductor will give a reminder once 5 minutes have passed, so that you answer all questions. We want to examine which functions in the tool you used. Please select the functions you used.

	Yes	Did not find/use this function
**I clicked on and watched the "tutorial".**	◯	◯
**I shuffled the order for the treatments in the tool**	◯	◯
**I removed outcomes from the table that I was not interested in**	◯	◯
**I clicked the box named "color guide"**	◯	◯
**I clicked the box named "practical issues"**	◯	◯

I used the tool so that I viewed a window similar to the one below.

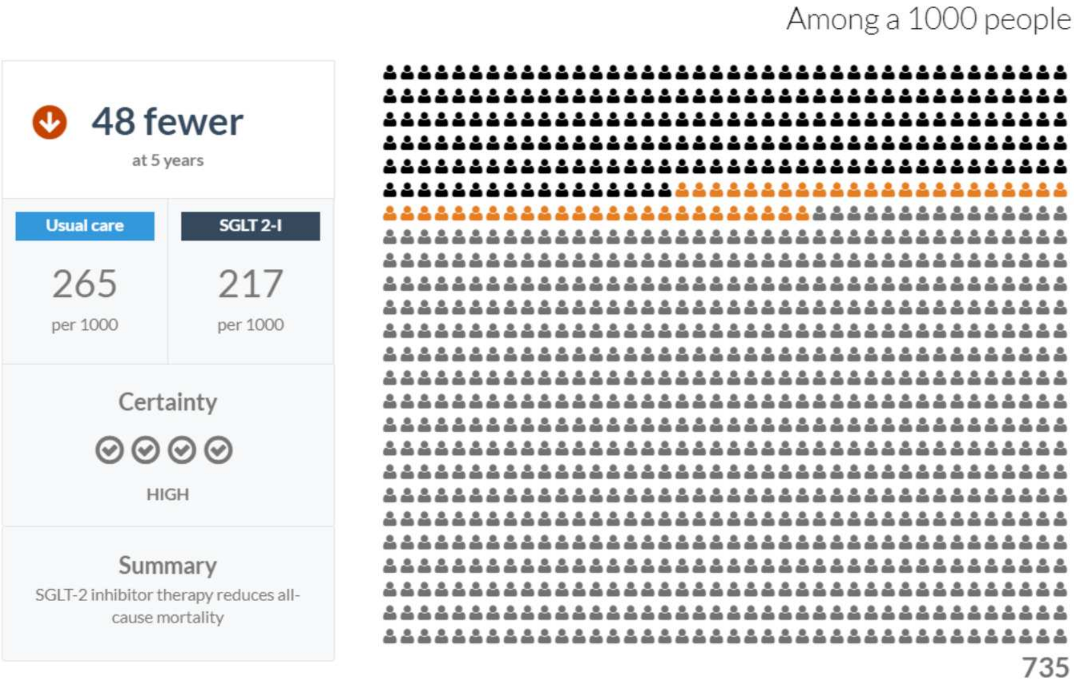

To what degree did the colour coding aid you in understanding the information in the tool?

- O Not at all
- O A little
- O Either or
- O Somewhat
- O Highly

You have up until now used version A/B of the tool. We would however, like for you to use and familiarize yourself with version A/B as well. Please press the link to use version A/B of the tool, and advance in the questionnaire once you feel like you have gotten to know the tool. Link:

MATCH-IT A: https://matchit.magicevidence.org/210104dist-diabetes-sof-open

MATCH-IT B: https://matchit.magicevidence.org/210104dist-diabetes-sof-closed

Preferences

You have now used two different versions of the MATCH-IT tool. First version A/B and then version A/B. Vi will now ask you some questions about your possible preferences for either one or the other version of the tool, relating to its use in finding the best treatment in the clinical scenario,

Do you prefer having the table open upon entry (version A) or choosing which outcomes to view on your own (version B)?

- o Strong preference for A
- o Preference for A
- o Slight preference for A
- o No preference
- o Slight preference for B
- o Preference for B
- o Strong preference for ¨

Do you prefer color coding (version A) or no color coding (version B)?

- o Strong preference for A
- o Preference for A
- o Slight preference for A
- o No preference
- o Slight preference for B
- o Preference for B
- o Strong preference for B

Overall, do you have a preference for version A or version B?

- o Strong preference for A
- o Preference for A
- o Slight preference for A
- o No preference
- o Slight preference for B
- o Preference for B
- o Strong preference for B

Thank you for your participation!
